# Supplementary material for: Markers of NETosis in Patients with Systemic Lupus Erythematosus and Antiphospholipid Syndrome
Source: Int J Mol Sci. 2023 May 24;24(11):9210. doi: 10.3390/ijms24119210 (PMC10252548; doi:10.3390/ijms24119210)
Supplement: Supplementary file 1 [file ijms-24-09210-s001.zip › ijms-2379824-supplementary.pdf]

Table S1. Characteristics of included patients

Parameters	SLE without APS n=47	SLE with APS n=30	PAPS n=41	HC n=20	*p*
Age, years, M± σ	34±9.84	39±8.27	44±10.55	31.5±9.71	*p* < 0.0001
Gender: female/male n, %	26 /4	34 /13	29 /12	14 /6	*p* = 0.394
Disease duration, years, Me [25; 75]	4,8 [0.7;11,1]	14 [10;22,5]	8,5 [2;15]	-	*p* < 0.0001

Note: SLE, Systemic lupus erythematosus; APS, Antiphospholipid syndrome; PAPS, primary antiphospholipid syndrome; HC, healthy controls. Me, median with an interquartile range; M±σ, where M is mean, σ is standard deviation; *p*, probability.

Table S2. Clinical and laboratory manifestations of SLE for the entire period of the disease according to the 1997 ACR criteria and ongoing therapy

Parameters	SLE without APS (n=30), n (%)	SLE with APS (n=47), n (%)	Total (n=77), n (%)
Malar rash	17 (57)	19 (40)	36 (47)
Discoid rash	2 (7)	-	2 (3)
Photosensitivity	14 (47)	12 (26)	26 (33)
Oral ulcers	11 (37)	7 (15)	18 (23)
Arthritis	24 (80)	31 (66)	55 (71)
Serositis	21 (70)	27 (57)	48 (62)
Renal disorder	19 (63)	18 (38)	37 (48)
Central nervous system damage	4 (13)	11 (23)	15 (20)
Hematological disorders	21 (70)	34 (72)	55 (71)
Immunological disorders	30 (100)	47 (100)	77 (100)
Positive antinuclear factor	30 (100)	47 (100)	77 (100)
SLEDAI-2K, Me [25;75]	11,5 [8;19]	4 [2;7]	6 [3;13]
ID SLICC/ACR, Me [25;75]	0 [0;1]	3 [2;4]	2 [0;3]
Therapy * , n (%)	27 (90)	45 (96)	72 (94)
Glucocorticoids	27 (90)	43 (92)	70 (91)
Hydroxychloroquine	22 (73)	37 (72)	59 (77)
DMARDs	11 (28)	6 (13)	17 (22)
- MMF	-6	-5	-11
- CYC	-4	-0	-4
- AZA	-1	-1	-2
Biologic DMARDs	3 (10)	10 (21)	13(17)
-RTX	-2	-10	-12
-BLM	-1	0	-1
-Targeted synthetic -DMARDs	1	-	1
-Baricitinib	1		1

Note: SLE, systemic lupus erythematosus; ACR, American College of Rheumatology, APS, antiphospholipid syndrome; SLEDAI-2K, Systemic Lupus Erythematosus Disease Activity Index; DI SLICC/ACR, The Systemic Lupus International Collaborating Clinics/ American College of Rheumatology Damage Index; DMARDs, disease-modifying antirheumatic drugs; MMF, mycophenolate mofetil; AZA, azathioprine; CYC, cyclophosphamide; RTX, rituximab; BLM, belimumab. *at the time of inclusion in the study.

Table S3. Clinical and laboratory manifestations of APS and ongoing therapy

**Parameters**	**SLE with APS (n=47), n (%)**	**PAPS (n=41), n (%)**	**Total (n=88), n (%)**
Thrombosis, n, %	41 (87)	34 (83)	75 (85)
Arterial, n (%)/N	8 (20)/41	7 (21)/34	15 (17)
Venous, n (%)/N	17(42)/41	14 (41)/34	31 (35)
Arterial+ Venous, n (%)/N	16 (39)/41	13 (38)/34	29 (33)
Pregnancy morbidity *, n (%)/N	17 (68)/25	15 (94)/16	32 (78)/41
≥3 unexplained consecutive spontaneous abortions before the 10th week of gestation	2 (12)/17	0	2 (5)/41
≥1 unexplained deaths of a morphologically normal fetus at or beyond the 10th week of gestation	4(24)/17	8(53)/15	12(29)/41
≥1 premature births of a morphologically normal neonate before the 34th week of gestation	1(6)/17	3(20)/15	4(10)/41
Early+late pregnancy loss	10(59)/17	4(27)/15	14(34)/41
aPL**			
Negative IgG aCL, n (%)	21 (45%)	11 (27%)	32 (36)
Low titers of IgG aCL, n (%)	2 (4%)	0	2 (2)
Medium titers of IgG aCL, n (%)	8 (17%)	6 (15%)	14 (16)
High titers of IgG aCL, n (%)	16 (34%)	24 (59%)	40 (45)
Negative IgM aCL, n (%)	39 (83)	33 (81)	70 (80)
Low titers of IgM aCL, n (%)	2 (4)	0	2 (2)
Medium titers of IgM aCL, n (%)	1 (2)	0	1 (1)
High titers of IgM aCL, n (%)	5 (11)	8 (20)	13 (15)
Negative IgG aβ2GP1 , n (%)	22 (47)	9 (22)	31 (35)

Low titers of IgG aβ2GP1, n (%)	3 (6)	5 (12)	8 (9)
Medium titers of IgG aβ2GP1, n (%)	7 (15)	3 (7)	10 (11)
High titers of IgG aβ2GP1, n (%)	15 (32)	24 (59)	39 (44)
Negative IgM aβ2GP1, n (%)	40 (85)	21 (78)	61 (69)
Low titers of IgM aβ2GP1, n (%)	1 (2)	1 (2)	2 (2)
Medium titers IgM aβ2GP1, n (%) of	4 (9)	3 (7)	7 (8)
High titers of IgM aβ2GP1, n (%)	2 (4)	5 (12)	7 (8)
Lupus anticoagulant in the medical history, n (%)	31 (70)/44	25 (69)/36	56 (64)
Therapy ***	45 (96)	40 (98)	85 (97)
Anticoagulants	36 (77)	37 (90)	73 (83)
Antiplatelet drugs	16 (34)	19 (46)	35 (40)

Note: SLE, systemic lupus erythematosus; APS, antiphospholipid syndrome; PAPS, primary antiphospholipid syndrome; aPL, antiphospholipid antibodies; aCL, anti- cardiolipin antibodies; IgG, immunoglobulin G; IgM, immunoglobulin M; aβ2GP1, antibodies to β2 glycoprotein 1; dsDNA antibodies, antibodies to double-stranded DNA; Me, median with an interquartile range; *Pregnancy and pregnancy morbidity during illness were considered. **aPL levels at the time of blood sampling; ***Therapies administered at the time of blood sampling.
